# Elevated H3K18 acetylation in airway epithelial cells of asthmatic subjects

**DOI:** 10.1186/s12931-015-0254-y

**Published:** 2015-08-05

**Authors:** Dorota Stefanowicz, Ja Young Lee, Kevin Lee, Furquan Shaheen, Hyun-Kyoung Koo, Steven Booth, Darryl A. Knight, Tillie-Louise Hackett

**Affiliations:** UBC Centre for Heart Lung Innovation, St. Paul’s Hospital, 1081 Burrard Street, Vancouver, V6Z 1Y6 BC Canada; School of Biomedical Sciences and Pharmacy, Faculty of Health and Medicine, University of Newcastle, Callaghan, NSW Australia; Department of Anesthesiology, Pharmacology and Therapeutics, University of British Columbia, Vancouver, BC Canada

## Abstract

**Background:**

Epigenetic adjustments of the chromatin architecture through histone modifications are reactive to the environment and can establish chromatin states which are permissive or repressive to gene expression. Epigenetic regulation of gene expression is cell specific and therefore, it is important to understand its contribution to individual cellular responses in tissues like the airway epithelium which forms the mucosal barrier to the inhaled environment within the lung. The airway epithelium of asthmatics is abnormal with dysregulation of genes such as epidermal growth factor receptor (EGFR), the ΔN isoform of the transcription factor p63 (ΔNp63), and signal transducer and activator of transcription 6 (STAT6), integral to differentiation, proliferation, and inflammation. It is important to establish in diseases like asthma how histone modifications affect tissue responses such as proliferation and differentiation.

**Objectives:**

To characterize the global histone acetylation and methylation status in the epithelium of asthmatic compared to healthy subjects and to identify the impact of these variations on genes involved in epithelial functions.

**Methods:**

Whole lungs were obtained from healthy and asthmatic subjects (n = 6) from which airway epithelial cells (AECs) were isolated and airway sections were taken for analysis of histone lysine acetylation and methylation by immunohistochemistry. AECs were subjected to chromatin immunoprecipitation (ChIP) using anti-H3K18ac and anti-H3K4me2 antibodies followed by RT-PCR targeting ΔNp63, EGFR, and STAT6. AECs were also treated with TSA and changes in ΔNp63, EGFR, and STAT6 expression were determined.

**Results:**

We identified an increase in the acetylation of lysine 18 on histone 3 (H3K18ac) and trimethylation of lysine 9 on histone 3 (H3K9me3) in the airway epithelium of asthmatic compared to healthy subjects. We found increased association of H3K18ac around the transcription start site of ΔNp63, EGFR, and STAT6 in AECs of asthmatics. However, we were unable to modify the expression of these genes with the use of the HDAC inhibitor TSA in healthy subjects.

**Discussion:**

The airway epithelium from asthmatic subjects displays increased acetylation of H3K18 and association of this mark around the transcription start site of ΔNp63, EGFR, and STAT6. These findings suggest a complex interaction between histone modifications and gene regulation in asthma.

**Electronic supplementary material:**

The online version of this article (doi:10.1186/s12931-015-0254-y) contains supplementary material, which is available to authorized users.

## Introduction

The sequence of the human genome is essentially the same in all cells of the body within a specific individual, yet the epigenome differs from tissue to tissue [1]. The DNA of each cell is packaged into nucleosomes where 146 base pairs of DNA wrap around an octamer of histone proteins which contains two H2A, H2B, H3, and H4 core histones [2]. An important mechanism for altering the chromatin structure to regulate gene expression is the covalent modification of the amino acid residues of core histone N-terminal tails [3, 4]. The acetylation of lysine residues on histone tails, mediated by histone acetyltransferases (HATs), has been positively associated with gene transcription [3, 5]. Whereas histone deacetylases (HDACs) remove the acetyl mark from histone tails resulting in a repressive chromatin state [3]. In contrast to acetylation, methylation of histone tails can be both activating and suppressive of gene expression depending on the particular residue [3]. Methyl groups are added to lysine or arginine residues by histone methyltransferases (HMTs) and removed by histone demethylases (HDMs) [3, 5].

The role of epigenetics in the pathogenesis of asthma remains unclear with evidence of both altered HAT and HDAC activity and expression in the airways of asthmatics [6–9]. In bronchial biopsies from asthmatic subjects, HAT activity was shown to be elevated and HDAC activity reduced [6, 7]. Although research investigating the exact HDACs involved in asthma is inconsistent, expression of certain inflammatory genes has been associated with acetylation of histone lysine residues in an epithelial cell line [10]. Further, histone methylation is a key regulator of many genes involved in chronic inflammation and epithelial mesenchymal transition [11–14] and as such may also play a role in the regulation of epithelial genes in asthma.

Abnormal expression of genes involved in repair and inflammation has been reported in the epithelium of asthmatic patients. The expression of epidermal growth factor receptor (EGFR), important for migration, proliferation, and differentiation, all integral components of the repair process, is elevated in the epithelium of childhood-onset and adult asthmatics [15–18]. Further, the expression of EGFR is increased in both damaged and intact regions of the airway epithelium of asthmatic subjects [15, 16]. This overexpression of EGFR indicates either that there is an unresolved repair process or that the epithelium is locked in a repair phenotype [15] contributing to the inflammatory and remodeling processes. Furthermore, there is an increase in the number of basal cells expressing the transcription factor p63 in the asthmatic epithelium [19]. In particular, the ΔNp63 isoform is heavily expressed in the epithelium compared to the larger TAp63 isoform [20]. ΔNp63 is essential for differentiation, adhesion, and proliferation [21, 22], and thus, much like EGFR, is an important factor in epithelial repair [20, 23]. The increased number of cells expressing p63 in the asthmatic epithelium may signify an inappropriate differentiation program in asthma, which would directly affect epithelial homeostasis and repair [20]. Another transcription factor, signal transducer and activator of transcription 6 (STAT6), is also overexpressed in the epithelium of severe asthmatics [24, 25]. In the epithelium, signaling through STAT6 activates the chemokines CCL11 and CCL5 [26, 27], resulting in the recruitment of eosinophils, basophils, T cells, and leukocytes to the epithelium. This upregulation of STAT6 is believed to contribute to the chronic inflammation seen in the airway mucosa of asthmatic patients. Together these studies support the rationale that alterations in the inflammatory and repair mechanisms of the airway epithelium contribute to chronic inflammation and airway remodeling in asthma.

Further, as the airway epithelium is the first point of contact with inhaled environmental agents, it is well positioned for environmental factors to influence gene expression and ultimately susceptibility to disease through epigenetic modifications. The impact of altered histone modifications on the regulation of genes associated with repair and inflammation in the epithelium of asthmatic subjects is unclear. We therefore hypothesized that epigenetic changes including histone acetylation and methylation contribute to the abnormal expression of EGFR, ΔNp63, and STAT6 in the epithelium of asthmatic subjects and thus influence the disease pathogenesis. In this study we observed epigenetic differences in the epithelium of asthmatic as compared to healthy subjects. Specifically, we identified an increase in global H3K18ac and H3K9me3 in the airway epithelium of asthmatic subjects. We then found gene-specific alterations to the histone acetylation status of of ΔNp63, EGFR, and STAT6 in asthmatic airway epithelial cells, however, attempting to modulate the expression of these genes through exposure to the HDAC inhibitor TSA was unsuccessful.

## Methods

### Study subjects

Airway sections and AECs were obtained from non-transplantable human donor lungs from the International Institute for the Advancement of Medicine (Edison, NJ, USA). The study was approved by the Providence Research Ethics committee, University of British Columbia. AECs from asthmatic and healthy subjects were isolated by protease digestion as previously described [28, 29]. Briefly, airways were blunt-dissected and cut into 2-4 cm sections. Segments were washed in PBS and placed in Bronchial Epithelial Basal Media (BEBM; Lonza, Basel, Switzerland) containing Pronase for 16 h at 4 °C. Sections were then washed in BEBM to aid cell dissociation and the harvested cell suspension was passed through a 70 μm nylon mesh. Pronase was neutralized after which the cell suspension was collected and resuspended in BEGM containing an antibiotic and antimycotic (Gibco). Cells were grown in 6-well plates in culture at 37 °C in 95 % air and 5 % CO_2_ and used at passage 2 for all experiments. Donor demographics for samples used for immunohistochemistry, chromatin immunoprecipitation, and immunoblot are listed in Tables 1 and 2.

**Table 1:** Donor demographics for immunohistochemistry

Phenotype	n	Average age° (range)	Sex (M/F)
Asthma	6	16 (8-26)	3/3
Healthy	6	17.3 (4-24)	4/2

*M* male, *F* female

°There were no significant differences between age for disease groups; unpaired two-tailed *t*-test p = 0.77

**Table 2:** Donor demographics for chromatin immunoprecipitation and immunoblot

Phenotype	n	Average age° (range)	Sex (M/F)
Asthma	5	18.2 (11-25)	3/2
Healthy	5	16.4 (11-20)	3/2

*M* male, *F* female

°There were no significant differences between age for disease groups; unpaired two-tailed *t*-test p = 0.59

The human bronchial epithelial 16HBE14o- cell line was kindly provided by the Gruenert laboratory [30] and maintained in Dulbecco’s Modified Eagle Medium (DMEM, Gibco) containing 10 % Fetal Bovine Serum (FBS; Gibco) and an antibiotic and antimycotic (Gibco). Cells were grown in 6-well plates in a 37 °C humidified environment (95 % air/5 % CO_2_) and used at passage 4.

### Immunohistochemical staining and image analysis of airway sections

Formalin fixed, paraffin embedded airway tissue sections were used for immunohistochemical staining of histone modifications. Briefly, 5 μm sections of airway tissue were deparaffinized in xylene and then rehydrated with graded ethanol and processed for antigen retrieval. Primary antibodies listed in Additional file 1: Table S1 were diluted in the appropriate blocking serum (goat or horse) and incubated overnight at 4 °C. Sections were then incubated with a biotinylated goat anti-rabbit or horse anti-mouse secondary antibody (1:100, Vector Laboratories, Burlingame, CA, USA) for 60 min at room temperature. Signal was amplified with the addition of Streptavidin-HRP (Dako) and the chromogen 3,3-diaminobenzidine (Dako) was added for visualization of antigen staining and counterstained with Harris Hematoxylin Solution (Sigma, Oakville, ON, Canada). Slides were dehydrated with graded ethanol then coverslipped with Cytoseal 60 mounting medium (Richard-Allan Scientific, Kalamazoo, MI, USA). Tris-buffered saline was used for all washes and dilutions.

Five images were obtained from each donor airway using the Nikon Eclipse 700 (Nikon Instruments, Melville, NY, USA), a 60x objective, and SPOT Advanced software (Diagnostic Instruments, Sterling Heights, MI, USA). To quantify staining, color segmentation and point counting of nuclei were performed with ImagePro Plus software (Media Cybernetics, Rockville, MD, USA). For color segmentation, the same unit area was taken then the epithelium was traced manually for each image. Colors indicating positive staining were set at a threshold and a measurement of this area was obtained followed by a measurement of the total traced area of epithelium. These values were used to calculate % positive area of airway epithelium (area of positive stain/total epithelial area) as previously described [31, 32]. For point counting, all nuclei with positive staining were initially manually tagged with a marker followed by a second marker to identify all unstained nuclei. These values were then used to calculate % positive nuclei (total positive nuclei/total nuclei *100) for each image.

### Chromatin immunoprecipitation

AECs were grown in culture to 80 % confluence before extraction of samples for chromatin immunoprecipitation (ChIP) experiments. ChIP was performed using the EpiTect ChIP Kit (Qiagen) according to the manufacturer’s protocol. Chromatin was sheared using a Sonic Dismembrator Model 100 (Fisher Scientific) cycled at 30 s on, and 30 s off, for a total of 25 min. Sheared chromatin was immunoprecipitated with 2 μg of either H3K18ac (ab1191, Abcam), H3K4me2 (GAH-3203, Qiagen) or IgG (Qiagen) antibody. DNA, including input fraction, was purified using spin columns before undergoing PCR.

### Real time polymerase chain reaction

Purified ChIP DNA was analyzed by quantitative real time PCR for enrichment of target genes. RT2 SYBR Green qPCR Master Mix (330520, Qiagen), 10 μM primers, and ddH20 were mixed with immunoprecipitated or input DNA and analyzed in triplicate by the ViiA7 (Life Technologies). Primers used are listed in Additional file 2: Table S2 and were designed to target loci both upstream (I) and downstream (II) of the transcription start site (TSS) except ΔNp63 where a suitable downstream primer set could not be identified. In this case, two loci upstream of the ΔNp63 TSS were chosen instead. Cycle threshold (Ct) values were recorded and used to calculate % Input for each sample. Samples for which the threshold cycle exceeded 35 were omitted from analysis as these were considered unreliable readings.

As per manufacturer’s protocol, confirmation of immunoprecipitation specificity was demonstrated by analyzing % Input at genes known to be expressed (positive locus) and repressed (negative locus). Both H3K18ac and H3K4me2 had high % Input at the glyceraldehyde-3-phosphate dehydrogenase locus (GAPDH, transcriptionally active euchromatin), and low % Input at the spermidine/spermine N1-acetyltransferase family member 2 (SAT2, heterochromatin) and myogenic differentiation 1 loci (MYOD1, transcriptionally inactive euchromatin) indicating antibody specificity (Additional file 3: Figure S1).

### Trichostatin A treatment

AECs and 16HBE14o- cells were grown in 6-well plates to 80 % confluence. 16HBE14o- cells were treated for 24 h with control media or the HDAC inhibitor Trichostatin A (TSA, T8552, Sigma) at the following concentrations: 10, 100, 500, and 1000 ng/μl whereas AECs were treated with control media or TSA at 100 ng/μl.

### SDS-PAGE and immunoblot

Depending on the molecular weight of each protein of interest, a 10-18 % SDS-polyacrylamine gel was used to ensure proper separation of proteins during electrophoresis. Following incubation with the specific primary antibody (see Additional file 4: Table S3) overnight, membranes were incubated for 2 h with either goat anti-mouse IR-800 (Vector Laboratories) or goat anti-rabbit Alexa 680 (Invitrogen) secondary antibodies (1:2500 dilution). Imaging of the labeled membrane was performed on the LI-COR Odyssey system and Odyssey software 1.1 was used to perform densitometry (LI-COR Biotechnology, Lincoln, NE, USA).

Data for H3K18ac were normalized to total H3 and presented as fold change over untreated control. Data for ΔNp63 and STAT6 were normalized to β-tubulin whereas EGFR was normalized to HSP-90 to maintain resolution of the gel at a high molecular weight. All data are presented as fold change over untreated control.

### Statistics

A two-tailed unpaired *t*-test was performed to determine differences between asthmatic and healthy subjects for all experiments except for the TSA dose response data, for which a one-way ANOVA and a *post hoc* Dunnett’s multiple comparison test was performed. A p-value of less than 0.05 was deemed significant.

## Results

### Increased acetylation of lysine 18 on histone 3 in asthmatic airway epithelium

**Fig. 1:**
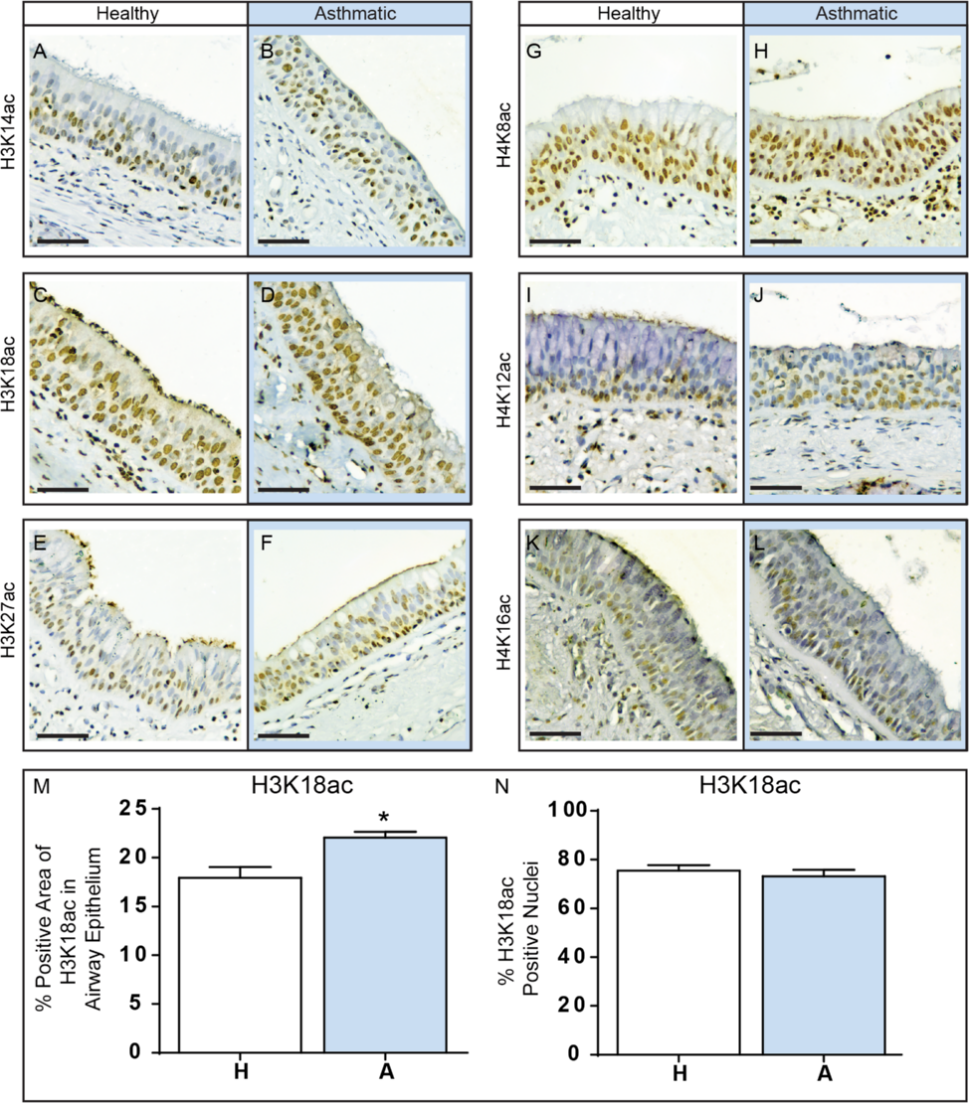
Acetylation of histone lysine residues in asthmatic and healthy airways. Airway sections from asthmatic and healthy patients were formalin fixed, embedded in paraffin and then sectioned for immunohistochemical analysis. Sections were stained for acetylation of H3K14ac (**a**, **b**), H3K18ac (**c**, **d**), H3K27ac (**e**, **f**), H4K8ac (**g**, **h**), H4K12ac (**i**, **j**) or H4K16ac (**k**, **l**). Scale bar is equal to 50 μm. The expression of and the % of positive nuclei stained for H3K18ac within the epithelium was quantified for both healthy (H, white bar) and asthmatic (A, blue bar) subjects (**m**, **n**). Data are expressed as % of positive area of airway epithelium ± SEM (n = 6) and % positive nuclei ± SEM (n = 6). A two-tailed unpaired *t*-test was performed, *indicates p < 0.05

To determine if there were differences in global histone acetylation between healthy and asthmatic epithelial tissue, we compared histone acetylation of 6 lysine residues on histones 3 and 4 (Fig. 1a-l). We identified a significant increase in % positive area of airway epithelium for acetylation of lysine 18 on histone 3 (H3K18ac) in asthmatic compared to healthy subjects (p = 0.02, Fig. 1m). However, when we counted the number of positive nuclei for H3K18ac, we found no difference between asthmatic and healthy epithelium (Fig. 1n). This suggests that more histones are acetylated at this particular residue in each positively stained cell within the epithelium from asthmatic subjects, which may affect the transcriptional activity of more genes as compared to healthy subjects. For the other acetylation marks, H3K14ac, H3K27ac, H4K8ac, H4K12ac, and H4K16ac, we found no difference in the % positive area or % positive nuclei in epithelium from asthmatic compared to healthy subjects (Additional file 5: Figure S2).

### Increased histone 3 lysine 9 trimethylation (H3K9me3) in asthmatic airway epithelium

**Fig. 2:**
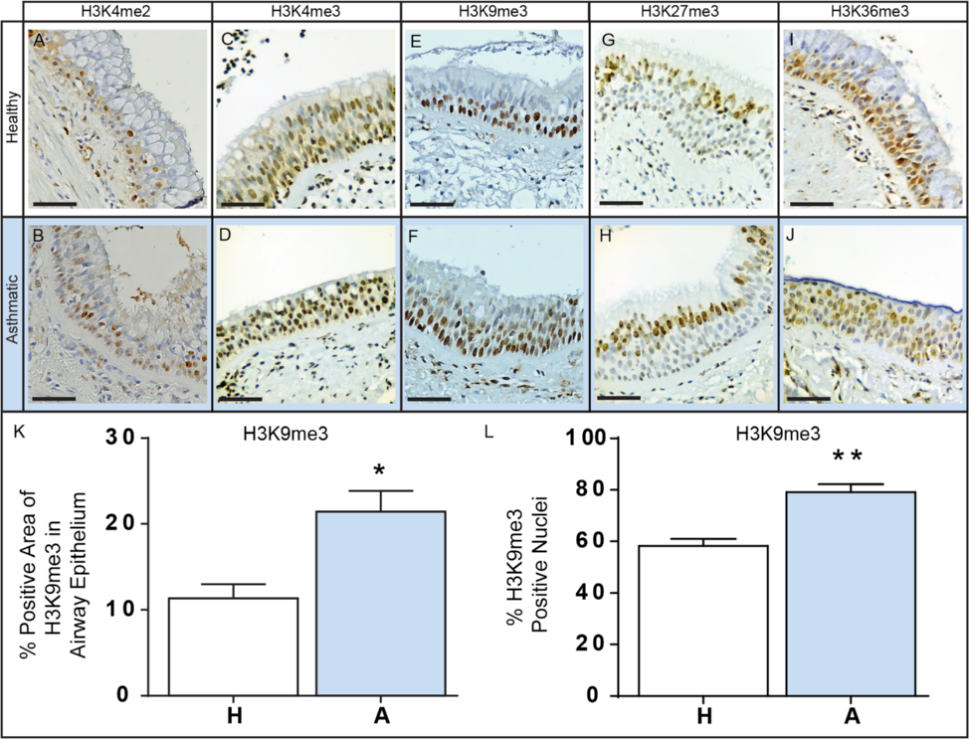
Methylation of histone lysine residues in asthmatic and healthy airways. Airway sections from asthmatic and healthy patients were formalin fixed, paraffin embedded and then sectioned for immunohistochemical analysis. Sections were stained for methylation of H3K4me2 (**a**, **b**), H3K4me3 (**c**, **d**), H3K9me3 (**e**, **f**), H3K27me3 (**g**, **h**), and H3K36me3 (**i**, **j**). Scale bar is equal to 50 μm. The expression of and the % of positive nuclei stained for H3K9me3 within the epithelium was quantified for both healthy (H, white bar) and asthmatic (A, blue bar) subjects (**k**, **l**). Data are expressed as % of positive area of airway epithelium ± SEM (n = 6) and % positive nuclei ± SEM (n = 6). A two-tailed unpaired *t*-test was performed, *indicates p < 0.05, **indicates p < 0.01

As histone methylation has well defined roles in regulating many genes, we next investigated whether global histone methylation differed between healthy and asthmatic epithelium. We found no difference in global histone methylation of the activating marks H3K4me2/me3, the silencing mark H3K27me3, nor the differentiation associated mark H3K36me3 (Fig. 2a-d,g-j, and Additional file 6: Figure S2A, C, E, and G). Additionally, we did not identify any differences in positively stained nuclei for these methylated histone lysine residues (Additional file 6: Figure S2B, D, F, and H). However, we did find a significant increase in the amount of staining in the airway epithelium for histone 3 lysine 9 trimethylation (H3K9me3) in asthmatic compared to healthy subjects (p = 0.015, Fig. 2e,f,k). This was mirrored by an increase in the number of nuclei positive for H3K9me3 in the epithelium from asthmatic subjects (p = 0.001, Fig. 2l). As we observed an increase in the % positive area and % positive nuclei of H3K9me3 in the epithelium of asthmatics, this could reflect that there are increased numbers of cells containing this repressive histone mark and not necessarily more of the H3K9me3 modification in each cell.

### Gene specific analysis of H3K18ac and H3K4me2 in asthmatic airway epithelial cells

Although our staining analysis was restricted to the airway epithelium our findings could also represent changes in histone modifications within dendritic and immune cells that may also be present within the epithelium and thus performing gene-specific experiments on epithelial cell cultures was necessary. We focused on H3K18ac, which had more staining per cell, as a potential modulator of genes overexpressed in the epithelium of asthmatics. Chromatin immunoprecipitation was preformed to identify whether the activating mark H3K18ac was enriched in regions surrounding the transcription start site (TSS) of ΔNp63, EGFR, and STAT6 as it has been shown that the acetylation of H3K18 is augmented predominantly around the TSS of active genes and not transcribed regions of genes [33]. As dysregulation of acetylation of the H3K18ac residue has been identified in other diseases to occur alongside H3K4me2 [34–38], we also investigated this activating epigenetic modification to determine if any changes were unique to H3K18ac or possibly part of a larger epigenetic phenomenon involving other histone modifications.

**Fig. 3:**
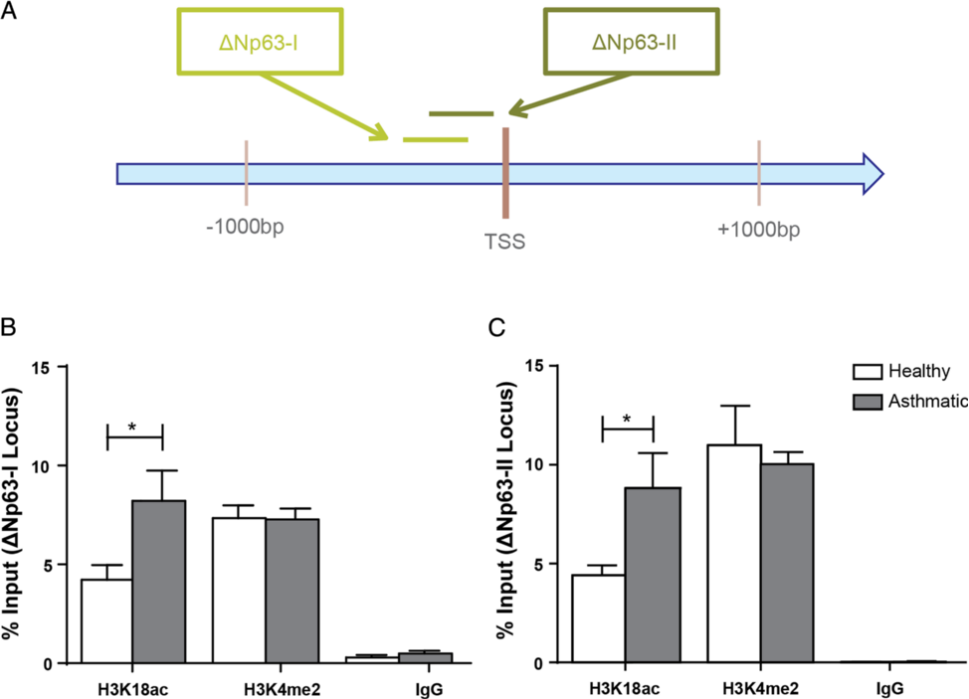
Association of H3K18ac and H3K4me2 at the ΔNp63 locus. Chromatin immunoprecipitation for H3K18ac, H3K4me2, and IgG was performed on AECs from asthmatic and healthy subjects. Primers located around the transcription start site (TSS) of ΔNp63 (**a**) were used for real time PCR targeting the ΔNp63-I (**b**) and the ΔNp63-II (**c**) loci. The presence of each histone modification and negative control was quantified and expressed as % Input at the target locus ± SEM (n = 5). A two-tailed unpaired *t*-test was performed to determine differences in % Input between asthmatic and healthy AECs for each locus, * indicates p < 0.05

As shown in Fig. 3a, we determined the association of H3K18ac at both the ΔNp63-I and ΔNp63-II loci. The % Input of H3K18ac at the ΔNp63-I locus in AECs from asthmatic donors was 8.21 % ± 1.54, whereas healthy donors showed 4.22 % ± 0.75 (p = 0.05, Fig. 3b). For ΔNp63-II, AECs derived from asthmatic subjects showed a % Input of 8.81 % ± 1.78 while healthy donors displayed 4.41 % ± 0.51 for H3K18ac (p = 0.04, Fig. 3c). However, we found no difference in binding of H3K4me2 at the ΔNp63-I or ΔNp63-II sites in AECs from asthmatic subjects compared to controls (Fig. 3b and c respectively).

**Fig. 4:**
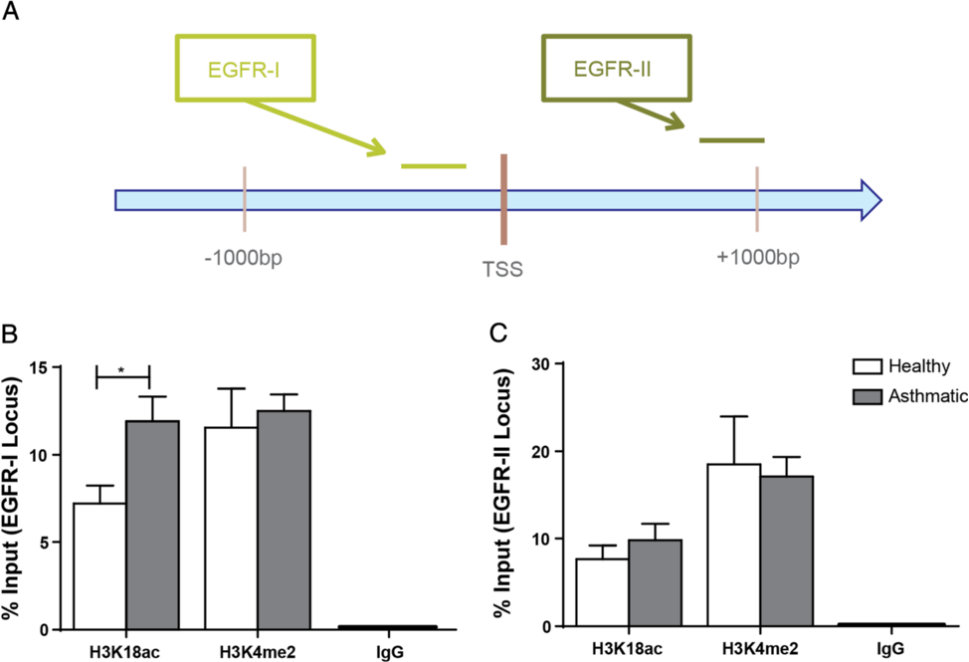
Association of H3K18ac and H3K4me2 at the EGFR locus. Chromatin immunoprecipitation for H3K18ac, H3K4me2, and IgG was performed on AECs from asthmatic and healthy subjects. Primers located around the transcription start site (TSS) of EGFR (**a**) were used for real time PCR targeting loci both (**b**) upstream (EGFR-I) and (**c**) downstream (EGFR-II) of the TSS. The presence of each histone modification and negative control was quantified and expressed as % Input at the target locus ± SEM (n = 5). A two-tailed unpaired *t*-test was performed to determine differences in % Input between asthmatic and healthy AECs for each locus, *indicates p < 0.05

Next, in Fig. 4 we investigated whether H3K18ac and H3K4me2 were differentially present surrounding the EGFR transcription start site (Fig. 4a) in AECs from healthy and asthmatic donors. The % Input of H3K18ac in asthmatic derived AECs was 11.92 % ± 0.95 as compared to 7.21 % ± 1.04 for healthy subjects at the EGFR-I locus (p = 0.03) but not for H3K4me2 (Fig. 4b). In contrast, we found no difference in association of H3K18ac or H3K4me2 between AECs from healthy or asthmatic subjects downstream of the EGFR TSS (Fig. 4c).

**Fig. 5:**
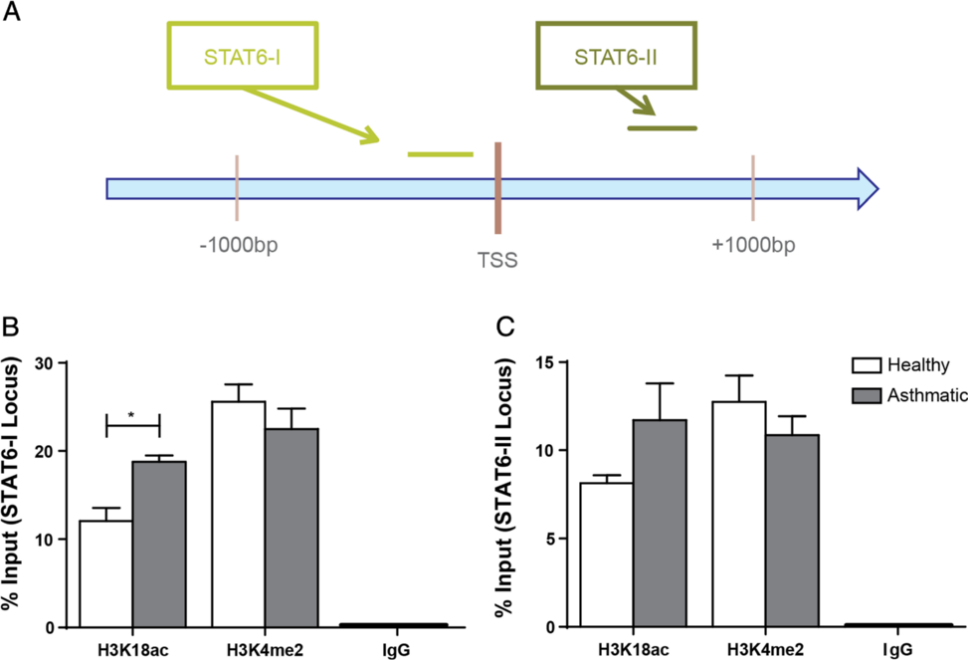
Association of H3K18ac and H3K4me2 at the STAT6 locus. Chromatin immunoprecipitation for H3K18ac, H3K4me2, and IgG was performed on AECs from asthmatic and healthy subjects. Primers located around the transcription start site (TSS) of STAT6 (**a**) were used for real time PCR targeting loci both (**b**) upstream (STAT6-I) and (**c**) downstream (STAT6-II) of the TSS. The presence of each histone modification and negative control was quantified and expressed as % Input at the target locus ± SEM (n = 5). A two-tailed unpaired *t*-test was performed to determine differences in % Input between asthmatic and healthy AECs for each locus, *indicates p < 0.05

Lastly in Fig. 5, we investigated the association of H3K18ac and H3K4me2 at the STAT6 TSS (Fig. 5a). In AECs from asthmatic subjects, the % Input of H3K4me2 was not different while the % Input of H3K18ac was greater than healthy subjects at the STAT6-I locus (18.76 % ± 0.73 versus 12.07 % ± 1.50, p = 0.004, Fig. 5b). We found no significant difference in binding of H3K18ac or H3K4me2 at the downstream STAT6-II locus in a comparison of healthy and asthmatic derived AECs (Fig. 5c).

### Modulation of ΔNp63, EGFR, and STAT6 with Trichostatin A

**Fig. 6:**
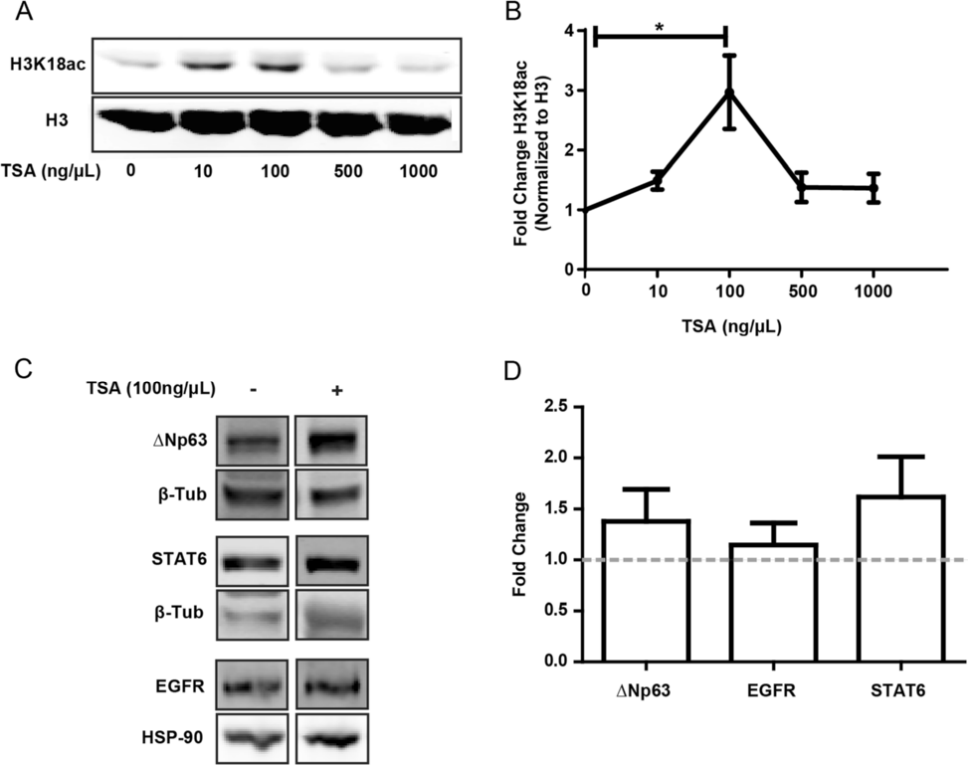
Modulation of H3K18ac, ΔNp63, EGFR, and STAT6 expression with TSA treatment. **a** and **b**: 16HBE14o- cells were treated with TSA at 0, 10, 100, 500, and 1000 ng/μl for 24 h. Protein was collected and SDS-PAGE immunoblot was performed to quantify H3K18ac and total H3. Data are presented as fold change H3K18ac normalized to H3 ± SEM (n = 3). A one-way ANOVA and a *post hoc* Dunnett’s multiple comparison test was performed. **c** and **d**: AECs from healthy subjects were treated with TSA (100 ng/μl) for 24 h. Protein was collected and SDS-PAGE immunoblot was performed to quantify the change in ΔNp63, EGFR, and STAT6 protein between control and TSA treated samples. Data are presented as fold change normalized to loading control (β-tubulin for ΔNp63 and STAT6, and hsp90 for EGFR) ± SEM (n = 5). A two-tailed paired *t*-test was performed for control versus treatment for each gene. *indicates p < 0.05

As the limited HAT inhibitors available exhibit low cell permeability and potency, we chose to investigate the functionality of acetylated H3K18ac in healthy AECs using the histone deacetylase inhibitor Trichostatin A (TSA). To ensure optimization, we first used the epithelial cell line 16HBE14o- and found that treatment of cells with 100 ng/μl TSA for 24 h resulted in significantly more H3K18 acetylation compared to baseline untreated cells (Fig. 6a and b). We next treated AECs from healthy donors with 100 ng/μl TSA for 24 h to determine if we could alter the expression of ΔNp63, EGFR, and STAT6 through changes in histone acetylation. Although there was an increase in protein expression of the three target genes ΔNp63, EGFR, and STAT6 in AECs induced by TSA, these changes were not statistically significant (Fig. 6c and d).

## Discussion

The goal of our study was to identify specific histone modifications that may affect the expression of ΔNp63, EGFR, and STAT6 which are known to be altered in the epithelium of asthmatic subjects. From a panel of 11 histone modifications, we identified an increase in H3K18ac and H3K9me3 within the airway epithelium of asthmatic compared to healthy subjects. As acetylation of histone lysine residues is generally associated with a permissive chromatin structure and gene expression, we directed our focus to H3K18 and found increased association of acetylated H3K18 around the transcription start site of ΔNp63, EGFR, and STAT6 in AECs derived from asthmatics when compared to healthy subjects. These data indicate that global changes in acetylated H3K18 may have important implications for defective epithelial repair in asthma.

Recently, Qian et al. identified the necessity of HDAC2 in the regulation of ΔNp63 expression [39]. This mechanism is thought to be dependent on the interaction between HDAC2 and the DEC1 transcription factor, which regulates ΔNp63 expression and has implications for growth suppression and cellular differentiation [39]. Although the authors did not clarify the exact epigenetic mechanism in terms of histone acetylation, they did find that treatment of mammary epithelial cell lines with the HDAC inhibitor TSA resulted in the induction of ΔNp63 [39]. We identified elevated association of H3K18ac at two regions upstream of the ΔNp63 TSS in AECs from asthmatic donors. ΔNp63 is the most prevalent p63 isoform expressed in human AECs with the ability to regulate multiple genes involved in epithelial functions such as proliferation and differentiation [20]. The finding of elevated histone acetylation at the ΔNp63 TSS provides insight into the possible mechanism of ΔNp63 gene regulation. As HAT activity is known to be increased in asthmatic epithelium [6, 7], it is possible that ΔNp63 may be a target of this epigenetic aberration.

For epigenetic regulation of EGFR expression, Chou et al. discovered a complex interaction between the histone deacetylase HDAC3, the histone acetyltransferase CBP, and the transcription factor SP1 [40]. Specifically, in human colon carcinoma cells, the authors showed that deacetylation of SP1 by HDAC3 results in increased binding of SP1 to the EGFR promoter [40]. The subsequent association of CBP with the SP1/HDAC3 complex results in histone acetylation and EGFR expression [40]. Of interest, the authors also suggested that priming of the EGFR gene was due to histone methylation, as they found enrichment of H3K4me2 at the EGFR promoter [40]. Another study in breast cancer epithelial cells found that EGFR is repressed by binding of the TIEG1/HDAC1 complex to SP1 sites on the EGFR promoter resulting in suppression of histone acetylation [41]. Our finding of increased association of acetylated H3K18 upstream of the EGFR TSS in AECs from asthmatics indicates that histone acetylation may be an epigenetic regulatory component of epithelial EGFR overexpression observed in asthma. Evidence of epigenetic regulation of EGFR has significant implications in the setting of asthma given its role in epithelial functions such as differentiation and proliferation and may provide a novel therapeutic modality in this disease.

STAT6 may interact with histone acetyltransferases and demethylases to facilitate transcriptional activation of STAT6 target genes [42–44] but the mechanism by which STAT6 itself is regulated by histone modification is not understood. Within the epithelium, STAT6 is an integral transcription factor in IL-13 mediated pathology and eotaxin gene activation [27, 45, 46]. Although the importance of STAT6 in the inflammatory process is recognized and STAT6 levels are elevated in the asthmatic epithelium [24], regulatory mechanisms controlling expression of this gene have yet to be elucidated. We identified elevated association of H3K18ac upstream of the STAT6 TSS in AECs from asthmatic compared to healthy donors. As histone acetylation is commonly associated with gene expression, our finding may provide an epigenetic mechanism for the increase in STAT6 expression seen in asthma.

Although differential levels of histone modifications may vary depending on specific disease pathology, there is growing evidence to support a mechanistic role for H3K18ac and H3K4me2 in cellular processes including differentiation. Specifically, low global cellular levels of these histone modifications have been identified in studies of prostate [36], pancreatic [37], kidney [38], and lung cancer [38] as predictors of poor prognosis. However, studies investigating global histone modifications in esophageal squamous cell carcinoma indicate that high global levels of H3K18ac and H3K4me2 are associated with tumor grade whereas low levels of H3K18ac are correlated to a better prognosis [35]. As tumor grade increases, carcinoma cells become more poorly differentiated [47], thus it is possible that the genes affected by H3K18ac and H3K4me2 in esophageal squamous cell carcinoma may contribute to halting the cellular differentiation program. Within our study, the % Input of H3K4me2 was not different between asthmatics and controls at the three genes examined, however, H3K4me2 was enriched at similar or higher amounts as H3K18ac at each site. This may support the theory that H3K4me2 primes the region for mechanisms, such as histone acetylation, which facilitate gene expression however further investigations are required to elucidate this interaction.

Due to the challenges with available HAT inhibitors and their use in cell culture, including low cell-permeability, low potency, and a lack of specificity [48], we focused on HDAC inhibition to drive ΔNp63, EGFR, and STAT6 gene expression. Since epithelial cells from healthy subjects displayed lower levels of H3K18ac at ΔNp63, EGFR, and STAT6, we hypothesized that exposure to the potent HDAC inhibitor TSA would increase the expression of these genes. Although TSA was able to induce H3K18 hyperacetylation in the 16HBE14o- cell line suggesting it was active, TSA exposure did not significantly increase the levels of ΔNp63, EGFR, or STAT6 in healthy AECs. These results could be due to the fact that TSA functions primarily on class I and class II but not class III deacetylases [49]. Recently, SIRT7 was identified as an H3K18ac specific class III histone deacetylase [50]. SIRT7 exerts its effects on genes which contain the ELK4 transcription factor binding site [50]. Interestingly, the ELK4 binding site has been identified close to the TSS of the STAT6 gene [50] and thus it is possible that deacetylation of H3K18 in at least one of the genes of interest is regulated by SIRT7. Another factor is that protein samples were collected 24 h post TSA treatment which may have been adequate time to induce alterations in histone acetylation, but not for the production of target proteins.

Numerous epigenetic mechanisms are now being proposed as pharmaceutical targets for various disease pathologies [51]. Such therapies that modify the epigenome are creating promising treatment options for diseases such as cancer, Huntington’s, and amyotrophic lateral sclerosis [52, 53]. In fact, the pan-HDAC inhibitors Vorinostat and Romidepsin have already been granted Food and Drug Administration approval for treatment of cutaneous T cell lymphoma [54, 55]. In terms of airway disease, the drug theophylline, which exerts it effects by activating HDAC2, is used to treat patients with corticosteroid resistant chronic obstructive pulmonary disease and has the potential to treat corticosteroid insensitive asthmatics [56–58]. When HDAC2 is allowed to function properly, it is recruited to inflammatory gene promoters by glucocorticoid receptors resulting in histone deacetylation and gene silencing [56, 57]. In this study we demonstrate increased H3K18 acetylation in the asthmatic epithelium. While the full clinical potential of epigenetic therapy has yet to be realized, for many histone modifications such as acetylation there are limited specific inhibitors. However, with a greater understanding of the role of epigenetics in the pathology of asthma, future work can be guided to develop specific epigenetic therapeutic options.

Although we show significant alterations in epithelial gene specific acetylation of H3K18 in asthma, our study does have limitations. Firstly, there is evidence that histone acetylation of inflammatory genes is responsive to corticosteroids [59], thus future studies stratifying the levels of H3K18ac by steroid use will be imperative to fully understand this epigenetic mechanism. In addition, the effects of HDAC inhibitors (including TSA) on inflammatory gene expression has been documented in numerous epithelial cell types [60]. Altered expression of important inflammatory genes in asthma pathogenesis such as interleukins and matrix metalloproteinases has been identified in response to TSA treatment [61–63]. As such, future work investigating the regulation of inflammatory genes through modulation of histone acetylation would be an exciting avenue to follow up. Secondly, we had to use donor lungs to obtain a sufficient quantity of primary AECs for the experiments conducted in this study and therefore have a relatively small sample size (n = 6 per group), thus further replication will be necessary. However, power calculations of the global increase in H3K18ac in asthmatic subjects show that this data is over 90 % powered to identify a significant effect. Lastly, we have not looked at other epigenetic mechanisms, such as DNA methylation, which may be important in the regulation of the genes examined in this study. Indeed, there is evidence of DNA methylation regulating the expression of EGFR, ΔNp63, and STAT6 in both human and animal studies [64–66]. However, previous work performed in our laboratory did not identify differential DNA methylation of EGFR in AECs from asthmatic, atopic or healthy subjects [67].

## Conclusions

In summary, we have identified elevated levels of acetylated lysine 18 on histone 3 in the epithelium of asthmatic as compared to healthy subjects. This does not exclude differences in various histone modifications at the gene-specific level, but it does provide a starting point for a possible epigenetic target for overexpressed genes in the airway epithelium of asthmatics. This is especially relevant as we identified elevated association of H3K18ac at the TSS of three genes which have been demonstrated to be dysregulated in the asthmatic epithelium, specifically ΔNp63, EGFR, and STAT6. However, the HDAC inhibitor TSA did not significantly alter the expression of these genes which may suggest a more complex mechanism of epigenetic control of gene expression in AECs. This study therefore highlights the need for development of more specific therapeutic epigenetic modifying agents to further understand the complexity of epigenetic modulators in asthma.

## Additional files

Additional file 1: Table S1. Antibodies used for immunohistochemical staining of donor airway tissue. GS indicates goat serum, HS indicates horse serum, TBS indicates Tris-Buffered Saline. (DOCX 12 kb) Additional file 2: Table S2. Primers used in real time PCR analysis of ChIP DNA. °Assay start position relative to transcription start site (TSS). (DOCX 11 kb) Additional file 3: Figure S1. Specificity of H3K18ac and H3K4me2 chromatin immunoprecipitation. Chromatin immunoprecipitation followed by real time PCR was used to show enrichment of activating marks H3K18ac and H3K4me2 at the GAPDH positive locus and low signal for both histone modifications at the MYOD1 and SAT2 negative loci. Data are presented as % Input at the target locus ± SEM (n = 10). (TIFF 107 kb) Additional file 4: Table S3. Antibodies used for immunoblot of cell protein lysate. (DOCX 11 kb) Additional file 5: Figure S2. Quantification of histone lysine acetylation in asthmatic and healthy airways. Airway sections from asthmatic and healthy patients were analyzed by immunohistochemistry for acetylated histone lysine residues. The expression of and amount of nuclei stained for H3K14ac (A, B), H3K27ac (C, D), H4K8ac (E, F), H4K12ac (G, H), and H4K16ac (I, J) within the epithelium was quantified for both healthy (H, white bar) and asthmatic (A, blue bar) subjects. Data are expressed as % of positive area of airway epithelium ± SEM (n = 6) and % positive nuclei ± SEM (n = 6). (TIFF 713 kb) Additional file 6: Figure S3. Quantification of histone lysine methylation in asthmatic and healthy airways. Airway sections from asthmatic and healthy patients were analyzed by immunohistochemistry for methylated histone lysine residues. The expression of and amount of nuclei stained for H3K4me2 (A, B), H3K4me3 (C, D), H3K27me3 (E, F), and H3K36me3 (G, H) within the epithelium were quantified for both healthy (H, white bar) and asthmatic (A, blue bar) subjects. Data are expressed as % of positive area of airway epithelium ± SEM (n = 6) and % positive nuclei ± SEM (n = 6). (TIFF 583 kb)
