# Supplementary material for: Elevated H3K18 acetylation in airway epithelial cells of asthmatic subjects
Source: Respir Res. 2015 Aug 5;16(1):95. doi: 10.1186/s12931-015-0254-y (PMC4531814; doi:10.1186/s12931-015-0254-y)
Supplement: Additional file 1: Table S1. — Antibodies used for immunohistochemical staining of donor airway tissue. GS indicates goat serum, HS indicates horse serum, TBS indicates Tris-Buffered Saline. (DOCX 12 kb) [file 12931_2015_254_MOESM1_ESM.docx]

**Additional file 1: Table S1. Antibodies used for immunohistochemical staining of donor airway tissue.**

| Epitope | Host | Company | Catalogue Number | Block | Primary Antibody Dilution |
| --- | --- | --- | --- | --- | --- |
| H3K14ac | Rabbit | Abcam | ab52946 | 50% GS | 1/100 in TBS + 25% GS |
| H3K18ac | Rabbit | Abcam | ab1191 | 50% GS | 1/200 in TBS + 25% GS |
| H3K27ac | Rabbit | Abcam | ab4729 | 50% GS | 1/400 in TBS + 25% GS |
| H4K8ac | Rabbit | Abcam | ab15823 | 100% GS | 1/700 in TBS + 50% GS |
| H4K12ac | Rabbit | Active Motif | 39165 | 100% GS | 1/400 in TBS + 50% GS |
| H4K16ac | Rabbit | Santa Cruz | sc-8662R | 10% GS | 1/100 in TBS + 5% GS |
| H3K4me3 | Rabbit | Abcam | ab8580 | 100% GS | 1/1200 in TBS + 50% GS |
| H3K4me2 | Rabbit | Abcam | ab7766 | 100% GS | 1/1500 in TBS + 50% GS |
| H3K9me3 | Rabbit | Abcam | ab8898 | 100% GS | 1/1500 in TBS + 50% GS |
| H3K27me3 | Mouse | Abcam | ab6002 | 50% HS | 1/100 in TBS + 25% HS |
| H3K36me3 | Rabbit | Abcam | ab9050 | 100% GS | 1/200 in TBS + 50% GS |
