# Supplementary material for: Elevated H3K18 acetylation in airway epithelial cells of asthmatic subjects
Source: Respir Res. 2015 Aug 5;16(1):95. doi: 10.1186/s12931-015-0254-y (PMC4531814; doi:10.1186/s12931-015-0254-y)
Supplement: Additional file 2: Table S2. — Primers used in real time PCR analysis of ChIP DNA. °Assay start position relative to transcription start site (TSS). (DOCX 11 kb) [file 12931_2015_254_MOESM2_ESM.docx]

**Additional file 2: Table S2. Primers used in real time PCR analysis of ChIP DNA.**

| ID | Gene | Source | Catalogue Number | Assay Startº | Forward Primer (5’-3’) | Reverse Primer (3’-5’) |
| --- | --- | --- | --- | --- | --- | --- |
| ΔNp63-I | p63 | In-house | - | -313 | TGTAAATCGTGGTGGTGGTG | GAGGCCTCTCCCATCTCATT |
| ΔNp63-II | p63 | In-house | - | -218 | CCTGTCTGTCTCCTGGGTTT | GAGGCGGGACTCTTCTCTTT |
| EGFR-I | EGFR | Qiagen | GPH1011923(-)01A | -393 | - | - |
| EGFR-II | EGFR | Qiagen | GPH1011923(+)01A | 932 | - | - |
| STAT6-I | STAT6 | Qiagen | GPH1017254(-)01A | -294 | - | - |
| STAT6-II | STAT6 | Qiagen | GPH1017254(+)01A | 608 | - | - |
