# Supplementary figures and images for: Elevated H3K18 acetylation in airway epithelial cells of asthmatic subjects
Source: Respir Res. 2015 Aug 5;16(1):95. doi: 10.1186/s12931-015-0254-y (PMC4531814; doi:10.1186/s12931-015-0254-y)

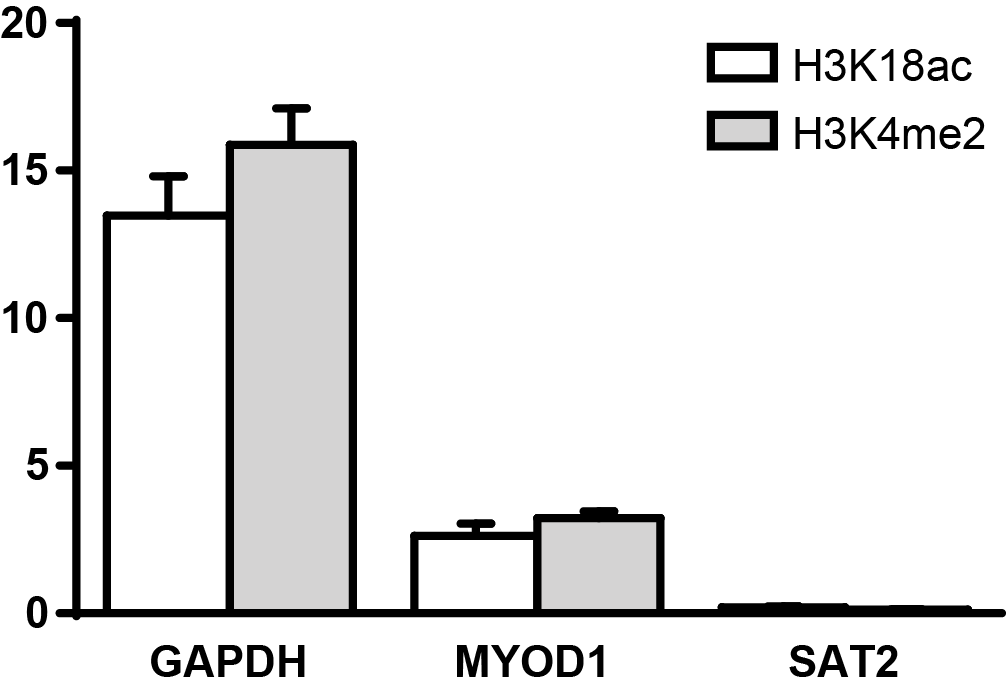

Supplement: Additional file 3: Figure S1. — Specificity of H3K18ac and H3K4me2 chromatin immunoprecipitation. Chromatin immunoprecipitation followed by real time PCR was used to show enrichment of activating marks H3K18ac and H3K4me2 at the GAPDH positive locus and low signal for both histone modifications at the MYOD1 and SAT2 negative loci. Data are presented as % Input at the target locus ± SEM (n = 10). (TIFF 107 kb) [file 12931_2015_254_MOESM3_ESM.tif]

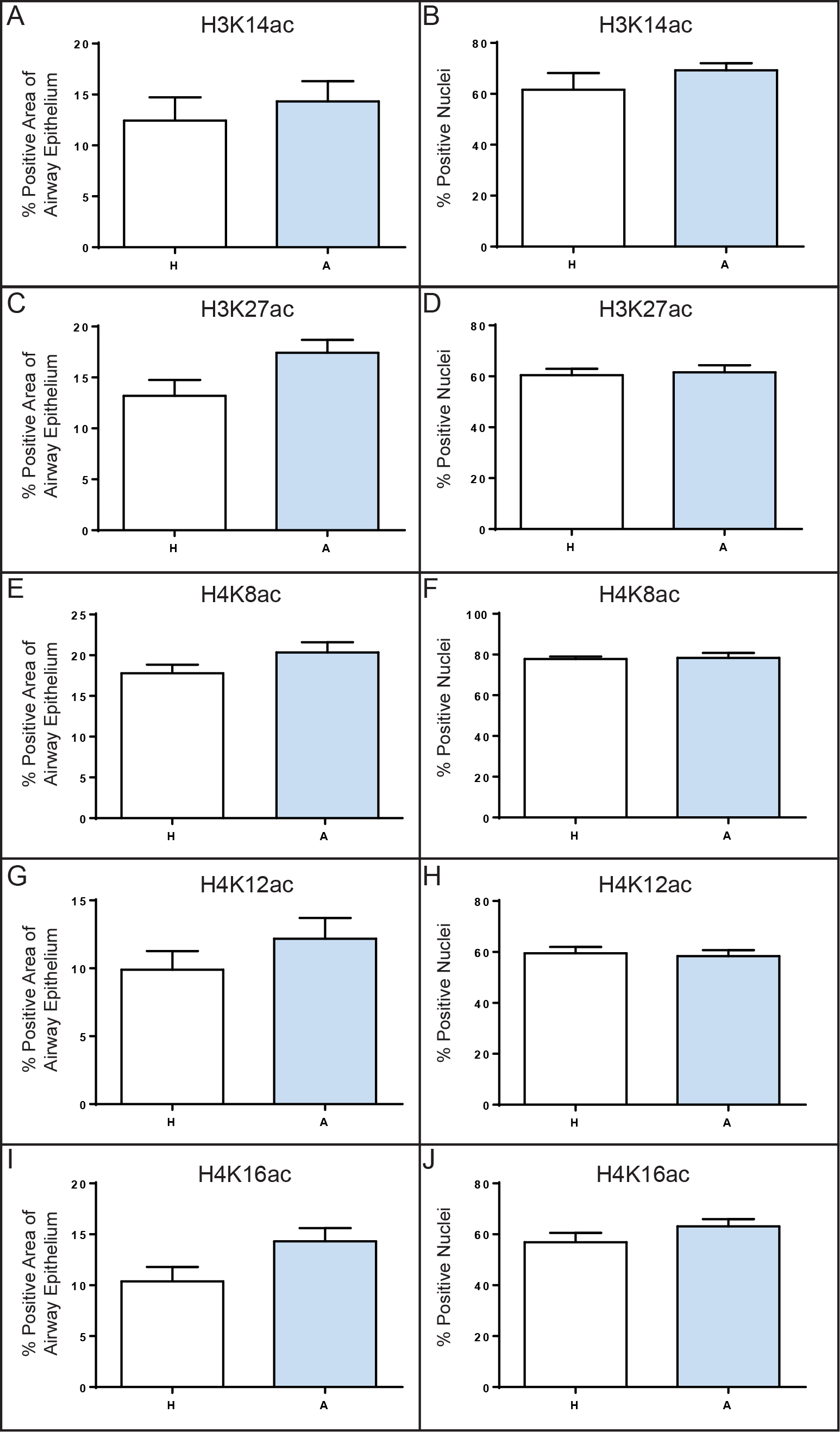

Supplement: Additional file 5: Figure S2. — Quantification of histone lysine acetylation in asthmatic and healthy airways. Airway sections from asthmatic and healthy patients were analyzed by immunohistochemistry for acetylated histone lysine residues. The expression of and amount of nuclei stained for H3K14ac (A, B), H3K27ac (C, D), H4K8ac (E, F), H4K12ac (G, H), and H4K16ac (I, J) within the epithelium was quantified for both healthy (H, white bar) and asthmatic (A, blue bar) subjects. Data are expressed as % of positive area of airway epithelium ± SEM (n = 6) and % positive nuclei ± SEM (n = 6). (TIFF 713 kb) [file 12931_2015_254_MOESM5_ESM.tif]

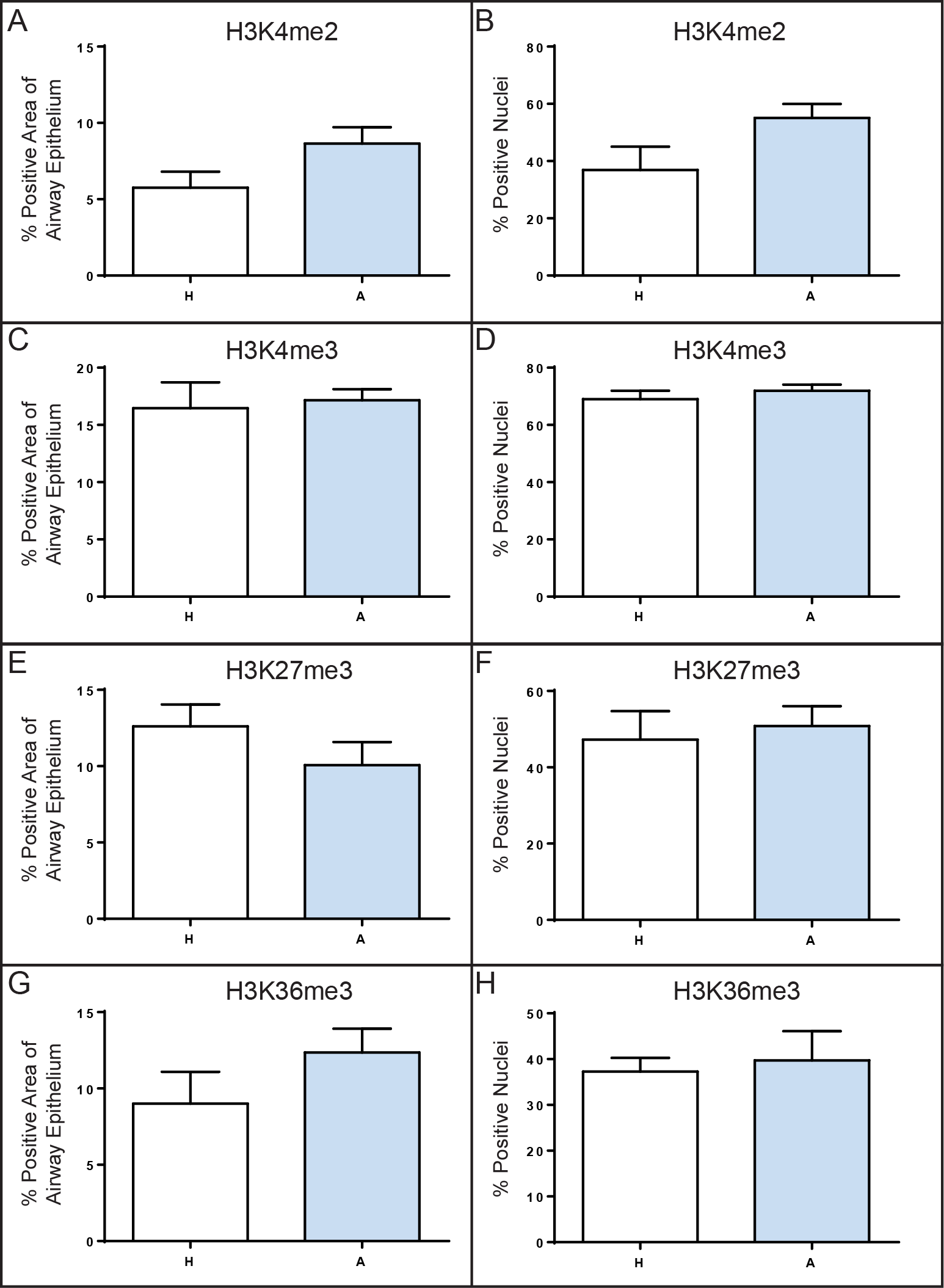

Supplement: Additional file 6: Figure S3. — Quantification of histone lysine methylation in asthmatic and healthy airways. Airway sections from asthmatic and healthy patients were analyzed by immunohistochemistry for methylated histone lysine residues. The expression of and amount of nuclei stained for H3K4me2 (A, B), H3K4me3 (C, D), H3K27me3 (E, F), and H3K36me3 (G, H) within the epithelium were quantified for both healthy (H, white bar) and asthmatic (A, blue bar) subjects. Data are expressed as % of positive area of airway epithelium ± SEM (n = 6) and % positive nuclei ± SEM (n = 6). (TIFF 583 kb) [file 12931_2015_254_MOESM6_ESM.tif]
