# Supplementary material for: Elevated H3K18 acetylation in airway epithelial cells of asthmatic subjects
Source: Respir Res. 2015 Aug 5;16(1):95. doi: 10.1186/s12931-015-0254-y (PMC4531814; doi:10.1186/s12931-015-0254-y)
Supplement: Additional file 4: Table S3. — Antibodies used for immunoblot of cell protein lysate. (DOCX 11 kb) [file 12931_2015_254_MOESM4_ESM.docx]

**Additional file 4: Table S3. Antibodies used for immunoblot of cell protein lysate.**

| Epitope | Host | Company | Catalogue Number | Primary Antibody Dilution |
| --- | --- | --- | --- | --- |
| H3 | Mouse | Abcam | ab10799 | 1/500 |
| H3K18ac | Rabbit | Abcam | ab1191 | 1/3000 |
| ΔNp63 | Mouse | Santa Cruz | sc-8341 | 1/500 |
| EGFR | Mouse | R&D Systems | 1095 | 1/500 |
| STAT6 | Rabbit | Epitomics | 1505-1 | 1/500 |
| β-tubulin | Mouse | Millipore | 05-661 | 1/2000 |
| HSP-90 | Mouse | BD Biosciences | 610418 | 1/1000 |
